# Gallbladder metastasis of renal clear cell carcinoma 15 years after primary cancer excision: a case report

**DOI:** 10.1186/s13256-018-1569-3

**Published:** 2018-05-31

**Authors:** Yasufumi Saito, Hiroshi Okuda, Makoto Yoshida, Seiji Okimasa, Toshikatsu Fukuda, Masatsugu Yano, Makoto Ochi, Yuzo Okamoto, Hirofumi Nakayama, Eiji Ono, Hideki Ohdan

**Affiliations:** 1 Department of Surgery, Medical Corporation JR Hiroshima Hospital, 3-1-36 Futabanosato, Higashi-ku, Hiroshima, 732-0057 Japan; 2 Department of Surgery, Onomichi General Hospital, Onomichi, Hiroshima Japan; 3 Department of Palliative Care, Medical Corporation JR Hiroshima Hospital, Hiroshima, Japan; 4 Department of Dialysis Surgery, Medical Corporation JR Hiroshima Hospital, Hiroshima, Japan; 5 Department of Pathology and Laboratory Medicine, Medical Corporation JR Hiroshima Hospital, Hiroshima, Japan; 6 Department of Gastroenterological and Transplant Surgery, Applied Life Sciences, Institute of Biomedical & Health Sciences, Hiroshima University, Hiroshima, Japan

**Keywords:** Renal cell carcinoma, Metastasis, Gallbladder

## Abstract

**Background:**

Renal cell carcinoma is well-known for its propensity to metastasize to unusual sites. However, metastasis to the gallbladder has been rarely reported in the literature.

**Case presentation:**

A 75-year-old Japanese (Asian) woman presented for further evaluation of a gallbladder polyp, 15 years after right radical nephrectomy for renal cell carcinoma. Computed tomography revealed a 12 mm enhancing pedunculated tumor in the gallbladder fundus. Open simple cholecystectomy was performed and the tumor was histologically confirmed as a metastasis of renal cell carcinoma to the gallbladder. Our patient is alive and has been disease-free for 3 years after cholecystectomy.

**Conclusions:**

Although metastasis of renal cell carcinoma is a rare differential diagnosis of gallbladder tumors, simple cholecystectomy is likely to offer a chance of long-term survival for patients with gallbladder metastases of renal cell carcinoma.

## Background

Metastasis to the gallbladder is rare and in most cases is found incidentally on autopsy [1, 2]. The rarity and clinical similarity of metastasis to the gallbladder to benign or other malignant gallbladder diseases make a correct diagnosis difficult in clinical practice.

Cancers of the kidney account for 4% of all newly diagnosed malignancies in men and 3% in women, and in most cases they are renal cell carcinomas (RCCs) [3]. Approximately one-third of patients with RCC already have metastases at the time of diagnosis, frequently to vascular-rich organs such as the lung, bone, and liver. Patients with distant metastases from RCC have a poor prognosis with a prospect of surviving for 5 years of < 10% [4]. However, curative resection of metastases in selected patients may improve long-term survival [5]. In patients with a solitary metastasis, a 35–50% prospect of surviving for 5 years after complete metastasectomy has been reported [4, 6].

We report a rare case of a patient with gallbladder metastasis from RCC diagnosed 15 years after primary cancer excision, and review the previously reported 38 cases. We discuss the condition’s presentation, surgical treatment, and survival outcomes. We also indicated the characteristics of preoperative image findings of gallbladder metastasis of RCC, which are considered to be helpful for the preoperative differential diagnosis of gallbladder tumors. It was thought to be clinically important that resection could allow long-term survival for patients with metachronous and localized RCC recurrences.

## Case presentation

A 75-year-old Japanese (Asian) woman underwent a right nephrectomy for RCC approximately 15 years ago. Our patient did not present symptoms at admission, and her past medical, social, family, and environmental history was not appreciable. Her occupation was home manager, and she was on no medication prior to diagnosis. She did not smoke and consume alcohol, and her temperature was 36.3 °C, her blood pressure was 122/82 mmHg, and her pulse was 68 per minute. Laboratory findings at admission are shown below. Her white blood cell count was 3800 × 10^3/^μL, red blood cell count 414 × 10^4^/μL, hemoglobin 13.0 g/dL, hematocrit 39.1%, platelets 19.6 × 104/μL, total bilirubin 0.7 mg/dL, direct bilirubin 0.2 mg/dL, aspartate transaminase 23 IU/L, alanine transaminase 9 IU/L, total protein 7.3 g/dL, albumin 4.5 g/dL, lactate dehydrogenase 188 IU/L, γ-glutamyltransferase 11 IU/L, alkaline phosphatase 201 IU/L, amylase 129 IU/L, blood urea nitrogen 13.6 mg/dL, creatinine 0.64 mg/dL, sodium 137 mEq/L, potassium 3.9 mEq/L, chlorine mEq/L, C-reactive protein 0.03 mg/dL, carcinoembryonic antigen 1.5 ng/mL, carbohydrate antigen 19–94 U/mL, urinalysis pH 7.0, no uric protein, no urinary sugar, no ketone body, no uric blood, no bilirubin, and no white blood cell. No microbial examination was performed.

The tumor was 9.1 × 7.8 cm in diameter and confined to the capsule of the kidney. Pulmonary lobectomy was performed for left lung metastasis 11 years after the primary resection and an additional lung partial resection was performed for the metachronous left lung metastasis 14 years after the primary resection.

**Fig. 1:**
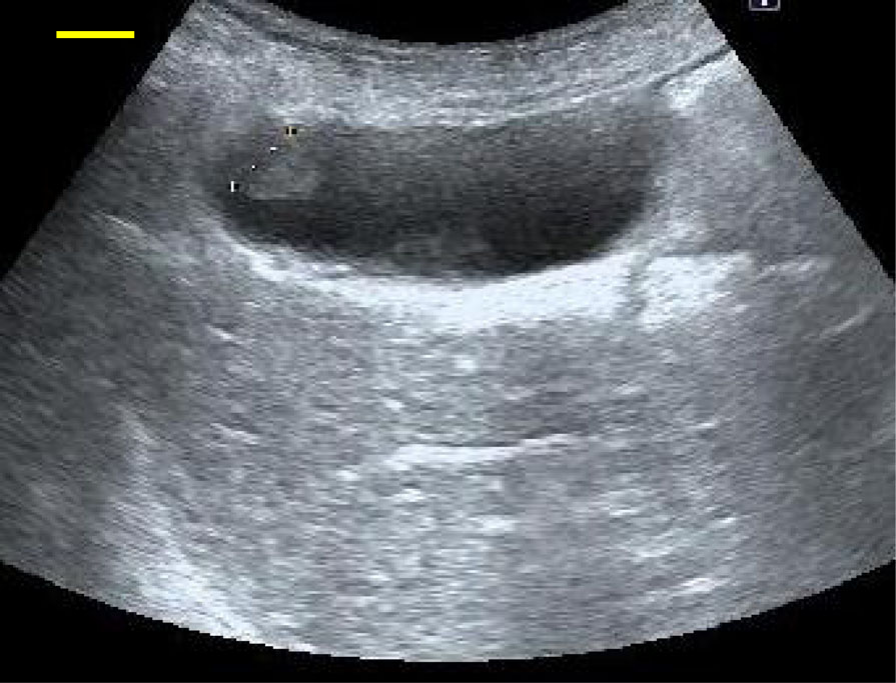
Ultrasonography shows an iso- to hyperechoic polyp at the fundus of the gallbladder (*yellow bar* indicates 10 mm)

Examinations including whole body computed tomography (CT) before each surgery demonstrated no evidence of distant metastasis. A surveillance follow-up CT scan revealed a gallbladder lesion. No symptoms suggested cholecystitis, and the only biochemical abnormality was a slight elevation in levels of aspartate transaminase and alanine transaminase. Ultrasonography (US) showed a mass at the gallbladder fundus. Its surface was smooth, and the inner echo was slightly high and homogenous (Fig. 1).

**Fig. 2:**
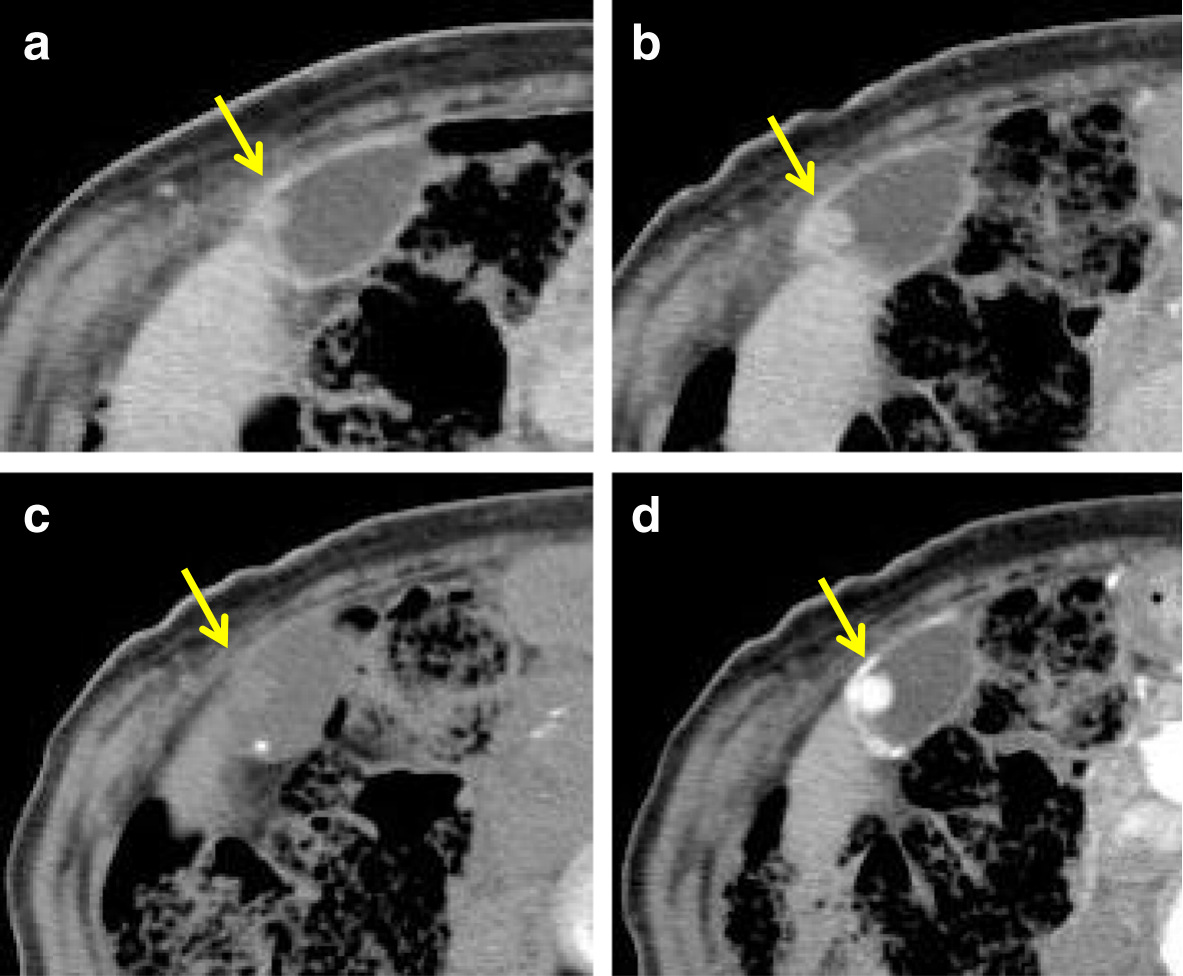
A computed tomography scan shows tumor growth from 4 mm to 12 mm over a span of 2 years (**a** 2 years prior, **b** present). A contrast-enhanced computed tomography scan shows high intensity of the tumor during the arterial phase (**d** *yellow arrow*) in comparison with plain computed tomography (**c** *yellow arrow*)

A contrast-enhanced CT scan showed a 12-mm polypoid mass with high attenuation, enlarged from 4 mm 2 years ago (Fig. 2a, b). It had significantly high intensity in the arterial phase. On the coronal reconstruction image, attenuation was inhomogeneous in the mass and relatively higher on the wall side (Fig. 2c, d). There was no significant accumulation of contrast agent in any organ other than the gallbladder.

Based on these image findings and the patient’s medical history, we initially thought the gallbladder mass was a malignant tumor such as a gallbladder carcinoma. The possibility that the tumor was metastatic cancer remained, and we therefore performed open approach cholecystectomy to confirm the diagnosis and perform adequate treatment.

**Fig. 3:**
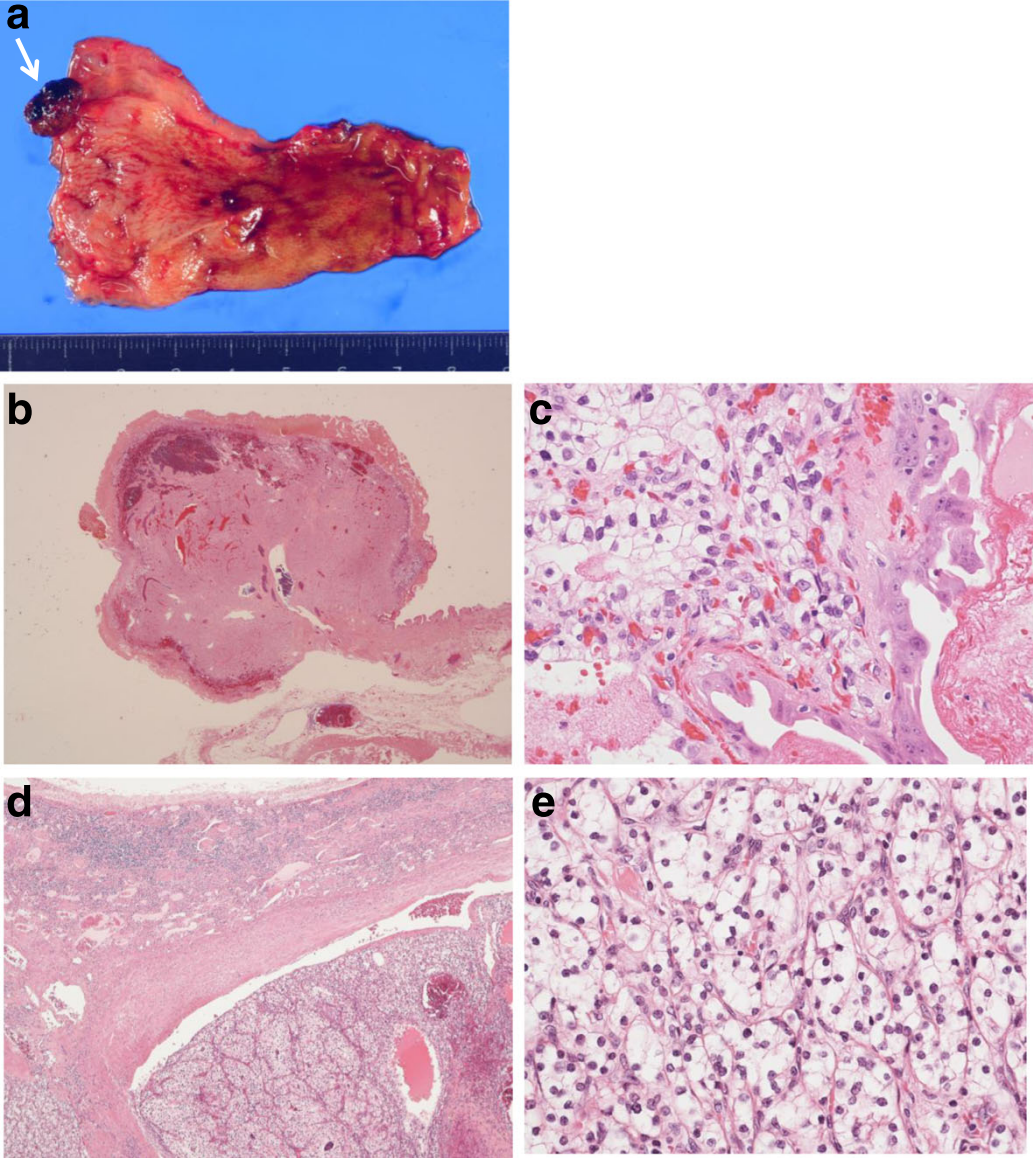
The surgical specimen shows a *black* pedunculated tumor in the fundus of the gallbladder (**a** *white arrow*). Pathological examination of hematoxylin and eosin staining shows tumor cells with clear cellular cytoplasm growth (**b** ×20, **c** ×200), and it is similar to the features of the renal primary tumor (**d** ×20, **e** ×200)

The isolated specimen showed a pedunculated tumor in the fundus of the gallbladder, and the surface of the tumor appeared black as a result of bleeding (Fig. 3a). Microscopically, we observed prominent vascular proliferation in the stalk and basal part of the tumor (Fig. 3b). The tumor was hypercellular and composed of clear cells arranged in funicular or alveolar growth with vascular interstitial tissue (Fig. 3c). The surface of the tumor was covered by epithelium, and extensive hemorrhage was observed under the surface (Fig. 3b). These histopathologic characteristics coincided with those of the renal tumor resected 15 years earlier (Fig. 3d, e).

**Fig. 4:**
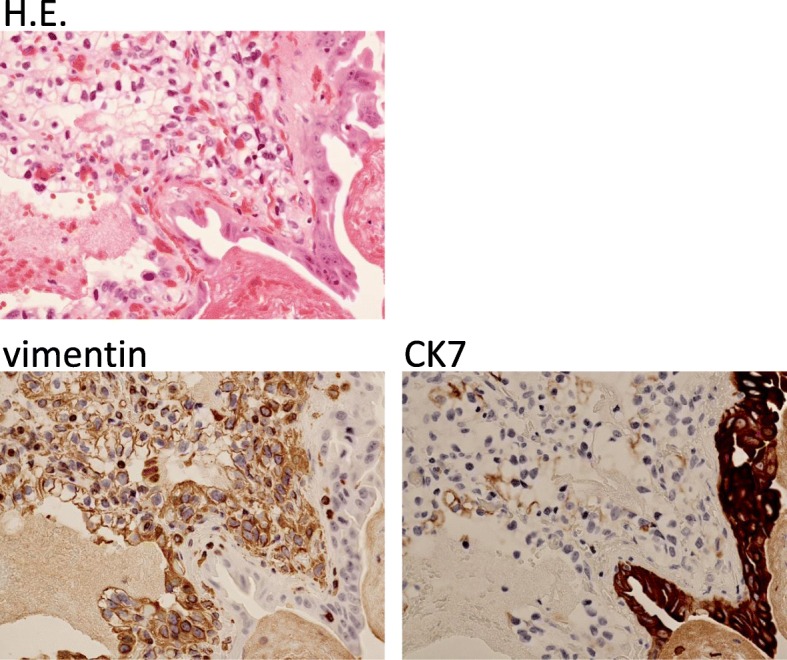
Immunohistochemistry staining for vimentin shows that the tumor cells stain strongly but the gallbladder epithelium is negative. CK7 immunohistochemical staining also shows that the tumor cells barely stain, but the gallbladder epithelium stains strongly

We performed immunohistochemical staining for vimentin and cytokeratin 7 (CK7). The tumor stained strongly for vimentin, but staining for CK7 was almost negative (Fig. 4), although for CK7, we observed a partially nonspecific immune reaction due to use of an automated immunostainer. These pathological features were similar to those of the renal primary tumor. Therefore, we diagnosed the gallbladder tumor as a metastasis from renal cell carcinoma. Our patient’s postoperative course was uneventful and she was discharged at postoperative day 5. She is alive and recurrence free 3 years after cholecystectomy.

## Discussion

This report showed a rare case of a patient who underwent open simple cholecystectomy and presented gallbladder metastasis from RCC. Even when compared with previous literature, the period from primary resection to gallbladder metastasis was relatively long. This observation shows that patients with metachronous and localized recurrences of RCC could be expected to achieve long-term survival following resection.

Distant metastases of RCC discovered during autopsy are found mostly in the lungs, liver, bones, and contralateral kidney. Metastasis to the gallbladder is very rare and is found in only 0.4–0.58% of autopsy cases due to RCC [2].

Malignant melanomas are the most common cause of metastatic tumors of the gallbladder in Western countries, and metastases from lung, renal, pancreatic, and colorectal cancers to the gallbladder have also been reported [2, 7]. To date, only 38 cases of RCC metastasizing to the gallbladder have been reported. Furthermore, there have only been seven cases, including this one, in which the metastasis was diagnosed 10 or more years after surgical resection of the RCC [3, 8–13]. In all these cases, the histological type was clear cell carcinoma.

On previous report,  the processes of metastasis to the gallbladder was grouped into two types, direct invasion of the tumor and invasion of the tumor into the capillaries, stating that the latter process is comparatively rare [2]. Gallbladder metastases occurred simultaneously in half of the cases and recurred metachronously in the other half. Another characteristic of these cases is the wide time range: 12 months to 27 years between the resection of the primary tumor and the reappearance of the tumor cells.

Dynamic contrast-enhanced CT is useful in the differential diagnosis of a metastatic gallbladder tumor from RCC and a primary gallbladder carcinoma, because the former is hypervascular [14, 15]. In the case we describe, contrast-enhanced CT showed the mass had high density on an arterial enhanced phase image. Another characteristic of these cases is the appearance of an echo-bright area on the surface of the tumor, indicating a submucosal tumor on US [16].

Only two reports have described the use of positron emission tomography (PET)/CT scans to differentiate gallbladder metastasis from RCC [2, 16]. Kawahara and colleagues documented a tumor mass on the gallbladder wall on PET/CT images without high accumulation of fluorodeoxyglucose (FDG) [2]. The role of PET/CT in gallbladder metastasis from RCC remains undefined.

Although RCC has some typical imaging characteristics, it remains extremely difficult to distinguish cases of primary and metastatic gallbladder carcinoma. In cases of RCC in which a gallbladder mass is observed simultaneously or metachronously, the possibility of gallbladder metastasis should be taken into account, although it is difficult to arrive at a preoperative diagnosis of gallbladder metastasis.

In the present patient, the gallbladder tumor was polypoid and over 10 mm in size. Furthermore, contrast-enhanced CT showed a high density on an arterial phase imaging. We thought it might be a gallbladder polyp or a malignant lesion, so we performed simple cholecystectomy for diagnosis and treatment since no suspicious findings of tumor invasion into the muscle layer of the gallbladder on preoperative imaging or intraoperative findings were noted.

Kavolius *et al.* have reported single organ metastasis and recurrence-free survival as prognostic factors after resection of metachronous metastatic lesions [5]. Chung *et al.* reported eight cases of patients with isolated gallbladder metastasis recurrence-free survival (observed median 1.1 years, range from 0.1 to 6 years) in a cohort study of 33 renal cell carcinoma cases [9], and they thought that isolated gallbladder single metastasis was an indication for surgery.

Although extended cholecystectomy is the standard operation when there is a strong suspicion of primary gallbladder cancer, it is important to excise metastatic lesions of RCC to the gallbladder. In Table 1, nine cases of simple cholecystectomy, including laparoscopic surgery, for an isolated metastasis to the gallbladder resulted in cancer-free survival in all cases (including the present case). Therefore, if there is no obvious invasion of the gallbladder bed, a simple resection including laparoscopic surgery is expected to be curative.

**Table 1:** Previously reported cases of metastatic renal cell carcinoma of the gallbladder. Permission was granted by Ishizawa T., Okuda J., Kawanishi T. et al. © Asian Surgical Association and published by Elsevier B.V. 2006 to reuse this table

Author	Age/Sex	Mode of metastasis	Syn or Meta	Interval from primary cancer	Other site of meta	Op procedure	Macroscopic findings	Size (cm)	Outcome	Year of source
Saito (present case)	75/F	Solitary	Meta	15 y	Lung	SC	Pedunculated	1.2×0.9	2 y alive	2018
Botting [18]	66/M	Solitary	Meta	1 y 7 m	(-)	SC	Polypoid	4.2×2	ND	1963
Terashima [19]	61/M	Multiple	Syn	(-)	Bone	EC	Mass	2×2	2 m death	1990
Satoh *et al.* [7]	71/M	Solitary	Syn	(-)	Pancreas	EC	Mushroom-shaped	4×2.5	1 y 7 m alive	1991
Fullarton [20]	43/F	Multiple	Syn	(-)	Pancreas, kidney	SC	Mass	3	5 m died from cancer	1991
Golbey [21]	84/M	Solitary	Meta	13y	(-)	SC	Pedunculated	3.5	ND	1991
Nagler [22]	82/M	Solitary	Meta	5y	(-)	EC	Polypoid	3×3	ND	1994
Pagano [23]	62/M	Solitary	Syn	(-)	Lung	SC	Round mass	3.5	disease free	1995
King [24]	64/M	Solitary	Syn	(-)	(-)	SC	Polypod	unclear	2 y 2 m disease free	1995
Fujii [25]	69/M	Multiple	Syn	(-)	Adrenal gland	EC	Polypoid	2.8×2.5	3 m disease free	1995
Coskun [26]	52/M	Multiple	Syn	(-)	bone	SC	Polypoid	3.5×2.5	ND	1995
Lombardo [27]	77/M	Solitary	Meta	5 y	(-)	EC	Polypoid	3×3	ND	1996
Kamimoto [28]	53/M	Multiple	Meta	4 y	(-)	LC	Polypoid	1.5	6 m alive	1996
Sparwasser [29]	46/M	Solitary	Meta	3 y 8 m	(-) Lung resected	SC	Polypoid	2.7×2.1	4 y4 m died from cancer	1997
Furukawa *et al.* [14]	41/M	Multiple	Syn	(-)	Lung, chest wall	SC	Pedunculated	1.9×1.3	ND	1997
Uchiyama [30]	64/M	Multiple	Meta	3 y	Kidney	SC	Pedunculated	1.9×1.1	7 m alive	1997
Celebi [31]	73/M	Solitary	Syn	(-)	Lung	EC	Mushroom-shaped	2.8×2	1 m died from other disease	1998
Ueki [32]	69/F	Solitary	Syn	(-)	(-)	EC	Pedunculated	1.6	7 m disease free	2001
Gekiya [33]	68/M	Solitary	Meta	15 y	(-)	SC	Polypoid	ND	1 y disease free	2002
Aoki [34]	63/M	Solitary	Meta	27 y	(-)	SC	Pedunculated	7.5×3	6 y disease free	2002
Aoki [34]	80/M	Solitary	Meta	8 y	Lung	SC	Pedunculated	4.5×2.5	2 y disease free	2002
Miyagi [35]	53/M	Solitary	Meta	10 y 6 m	(-)	LC	Polypoid	2.5×1.5	ND	2003
Limani [36]	64/M	Solitary	Meta	1 y	(-)	LC	Mass	ND	ND	2003
Ishizawa *et al*. [15]	73/M	Solitary	Meta	5 y	(-)	SC	Pedunculated	3.5×2	2 y disease free	2006
Hellenthal [37]	39/M	Solitary	Syn	(-)	(-)	SC	Polypoid	ND	2 y 6 m alive	2007
Ricci [9]	72/F	Solitary	Meta	16 y	Pancreas	LC	Mass	ND	ND	2008
Nojima [38]	61/M	Solitary	Syn	(-)	(-)	SC	Polypoid	1.5	10 m alive	2008
Sand [39]	48/F	Solitary	Meta	5 y	Pancreas, kidney	SC	ND	ND	2 m alive	2009
Patel *et al*. [1]	64/F	Solitary	Meta	6 y	(-)	LC	Polypoid	3	ND	2009
Kawahara *et al*. [2]	73/F	Solitary	Syn	(-)	Lung	SC	Polypoid	1.0×0.8	ND	2010
Shoji *et al*. [8]	50/M	Multiple	Meta	3 y	Adrenal grand	SC	Polypoid	1.1×0.9	8 m alive	2010
Fang *et al*. [13]	45/M	Solitary	Meta	1 y	Lung	SC	Polypoid	1.9×1.0	2 y 4 m death	2010
Fang *et al*. [13]	65/F	Solitary	Meta	1 y	Psoas muscle	SC	Polypoid	2.5×2.5	7 m death	2010
Fang *et al*. [13]	54/M	Solitary	Meta	7 y	(-)	SC	Polypoid	1.5×1.0	2 y 3 m alive	2010
Fang *et al*. [13]	51/M	Solitary	Meta	6 y	Kidney	SC	Polypoid	1.7×0.8	3 y 1 m alive	2010
Decoene *et al*. [10]	47/F	Solitary	Meta	16 y	Bone, ovary	LC	Polypoid	1.9	ND	2011
Jain and Chopra [12]	49/F	Solitary	Meta	6 y	(-)	SC	Polypoid	1.45×1.0	ND	2013
Ueda *et al*. [3]	43/M	Solitary	Meta	1 y	(-)	EC	Pedunculated	2.6	ND	2015

*Abbreviations*: *Syn* synchronous metastasis, *Meta* metachronous metastasis, *SC* simple cholecystectomy, *ND* not determined, *EC* extended cholecystectomy, *LC* laparoscopic cholecystectomy

For an adequate follow-up and informed decisions about adjuvant immunotherapy with interleukin-2 and interferon alpha after cholecystectomy, gallbladder metastasis of RCC should be differentiated from primary clear cell carcinoma of the gallbladder through histochemical examination. Immunohistochemically, primary clear cell carcinoma of the gallbladder is strongly positive for CK7 but negative for vimentin, and metastatic RCC of the gallbladder is positive for vimentin but negative for CK7 [17]. Based on the immunohistochemical findings, our final diagnosis was metastatic gallbladder tumor from RCC as opposed to primary clear cell carcinoma of the gallbladder.

The follow-up information on the previously reported cases is not sufficient to demonstrate the curability of cholecystectomy for a metastasis of RCC, since late recurrence is not uncommon with RCC. However, nine patients were reported to be cancer free with the longest follow-up interval of 6 years after cholecystectomy, and eight of these had a solitary metastasis. These reports suggest a favorable prognosis after cholecystectomy, particularly in patients with a solitary metastasis. Even for multiple metastases of RCC, cholecystectomy may be advocated, because the survival rates after curative resection of second and third metastases have not been found to be different from those after a first metastectomy [5].

## Conclusions

In conclusion, we describe a rare case of gallbladder metastasis from RCC diagnosed 15 years after primary cancer resection. In patients with a history of RCC, observation of a vascular-rich polypoid lesion of the gallbladder should raise the possibility of metastasis. Cholecystectomy may result in favorable long-term survival of patients with RCC metastases to the gallbladder.

## Abbreviations

CK7: Cytokeratin 7
CT: Computed tomography
FDG: Fluorodeoxyglucose
PET: Positron emission tomography
RCC: Renal cell carcinoma
US: Ultrasonography

## Authors’ information

YS, MY, TF, MY, and YO are Staff Surgeons of the Department of Surgery, Medical Corporation JR Hiroshima Hospital. HO is a Staff Surgeon of the Department of Surgery, Onomichi General Hospital. SO is a Staff Doctor in Palliative Care, Medical Corporation JR Hiroshima Hospital. MO is a Staff Member in Dialysis Surgery, Medical Corporation JR Hiroshima Hospital. HN is a Pathologist in the Department of Pathology and Laboratory Medicine, Medical Corporation JR Hiroshima Hospital. EO is the Hospital Director, Medical Corporation JR Hiroshima Hospital, and HO is a Professor of the Department of Gastroenterological and Transplant Surgery, Applied Life Sciences, Institute of Biomedical & Health Sciences, Hiroshima University.
